# Supplementary material for: Monitoring life expectancy levels during the COVID-19 pandemic: Example of the unequal impact of the first wave on Spanish regions
Source: PLoS One. 2020 Nov 5;15(11):e0241952. doi: 10.1371/journal.pone.0241952 (PMC7643983; doi:10.1371/journal.pone.0241952)

**Appendix I**

**Figure S1.** Weekly life expectancy at birth in Spain and its 17 regions by sex (weeks 1-27, 2019 and 2020)


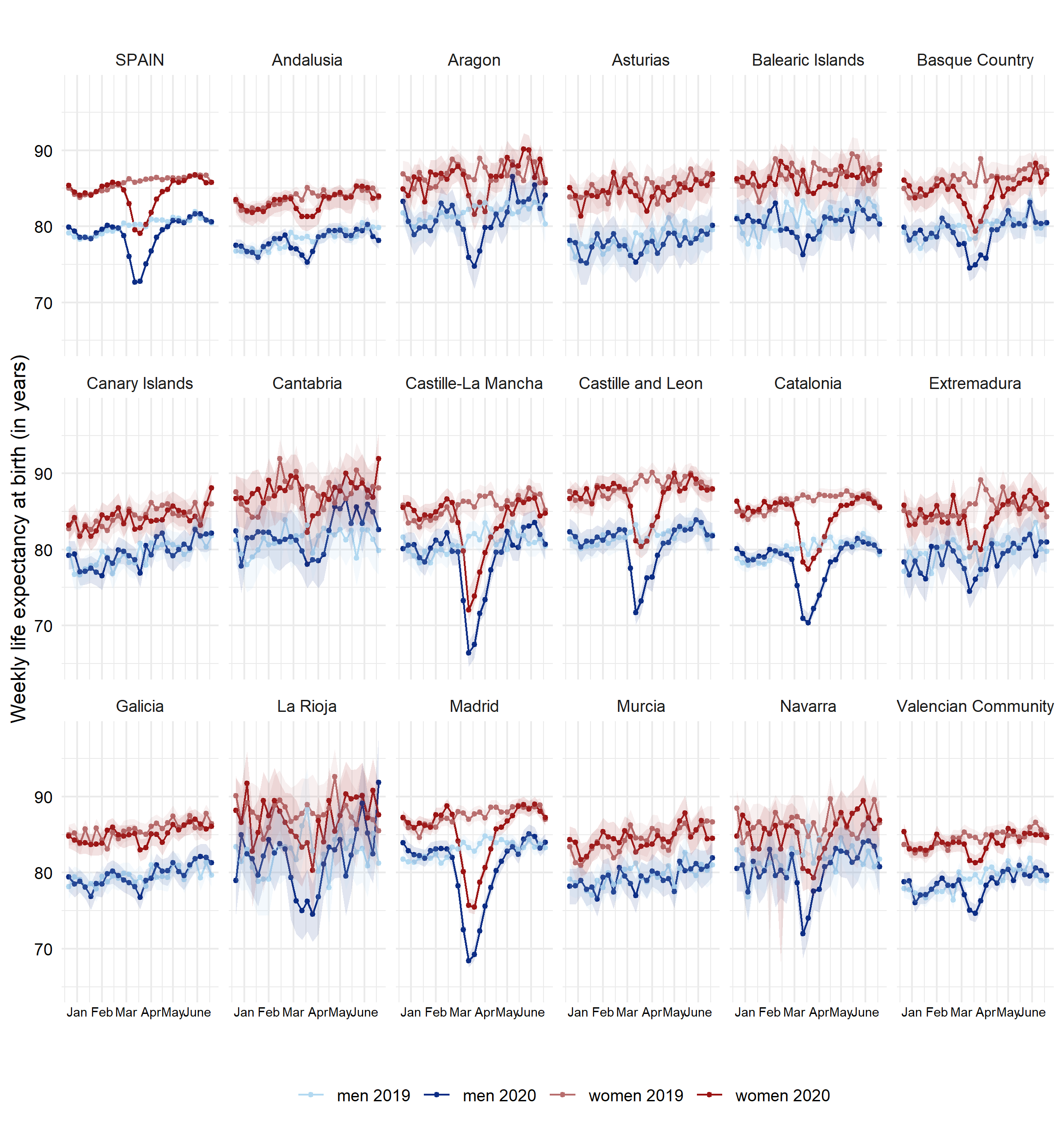


**Figure S2.** Average weekly life expectancy change in weeks 11-20, 2020 (March 9^th^ till May 17^th^) compared to the corresponding weeks in 2019.


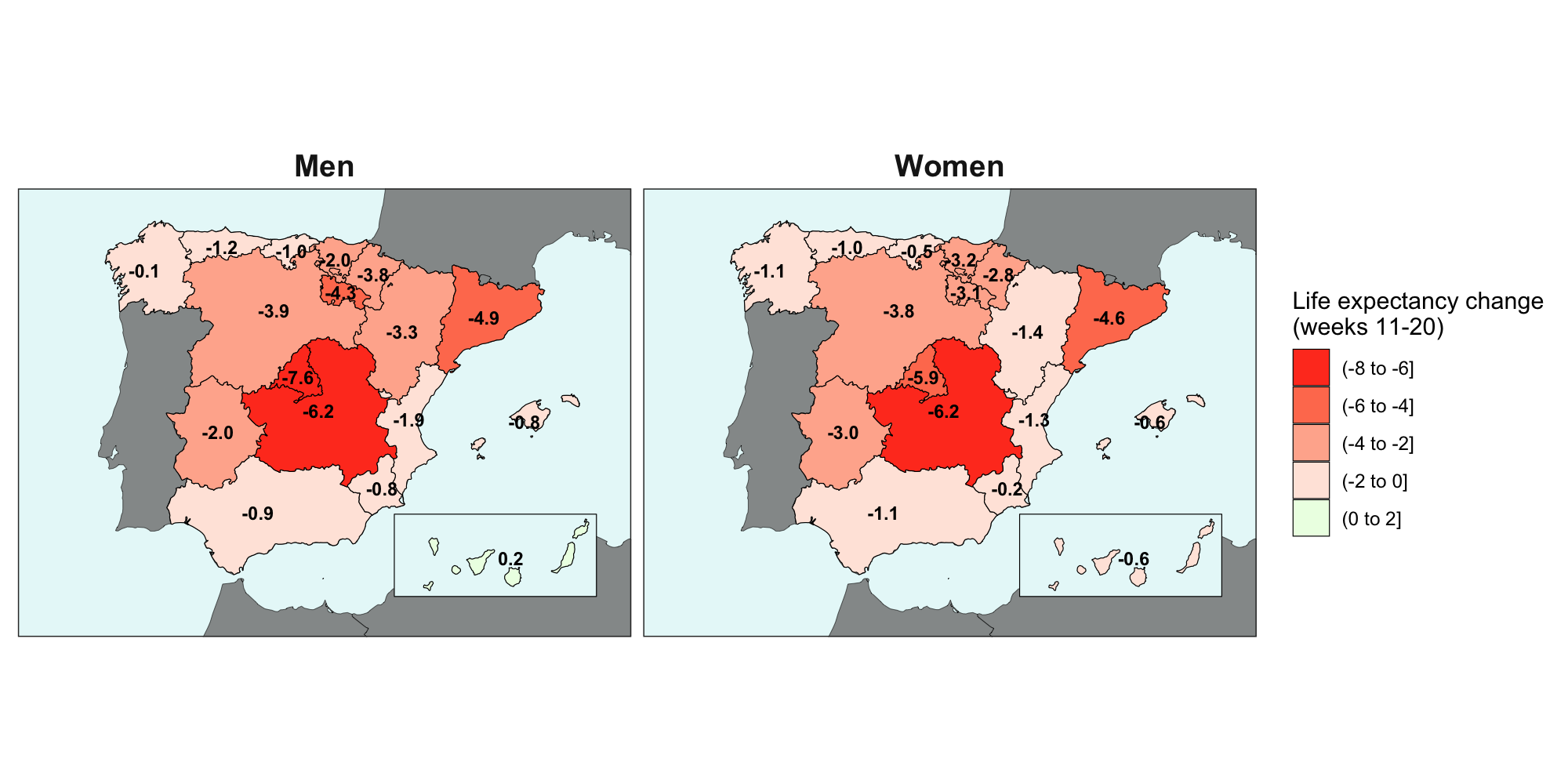


**Figure S3**. Annual life expectancy at ages 50 and 65 and 75 in 2019, 2020* and differences between periods for Spain and its 17 regions by sex


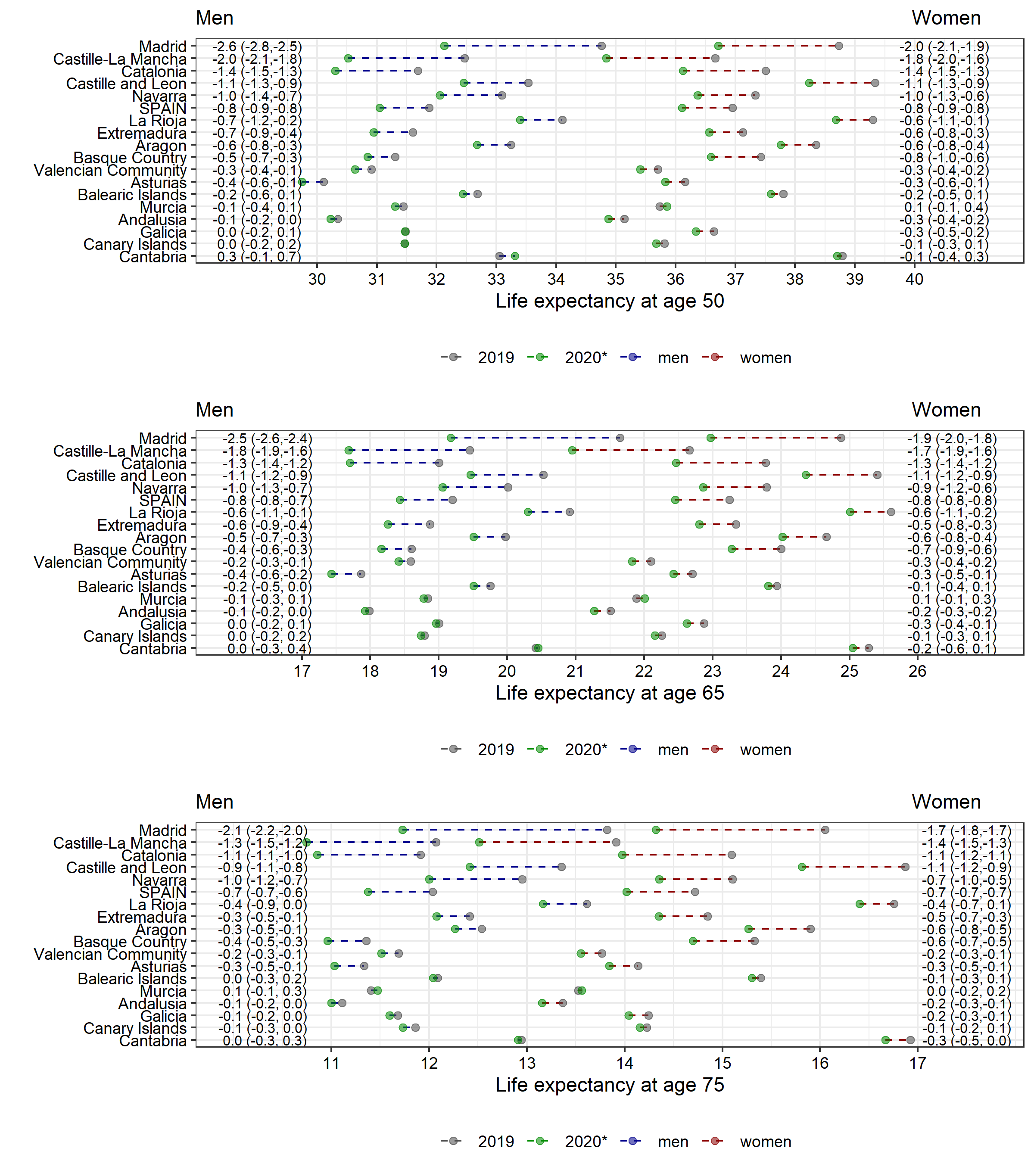
* Annual life expectancy at birth in 2020* was estimated using death counts from the shifted annual reference period up to 5 July 2020.

**Figure S4.** Weekly life expectancy at age 50 (with 95% confidence intervals) in Spain and its 17 regions by sex


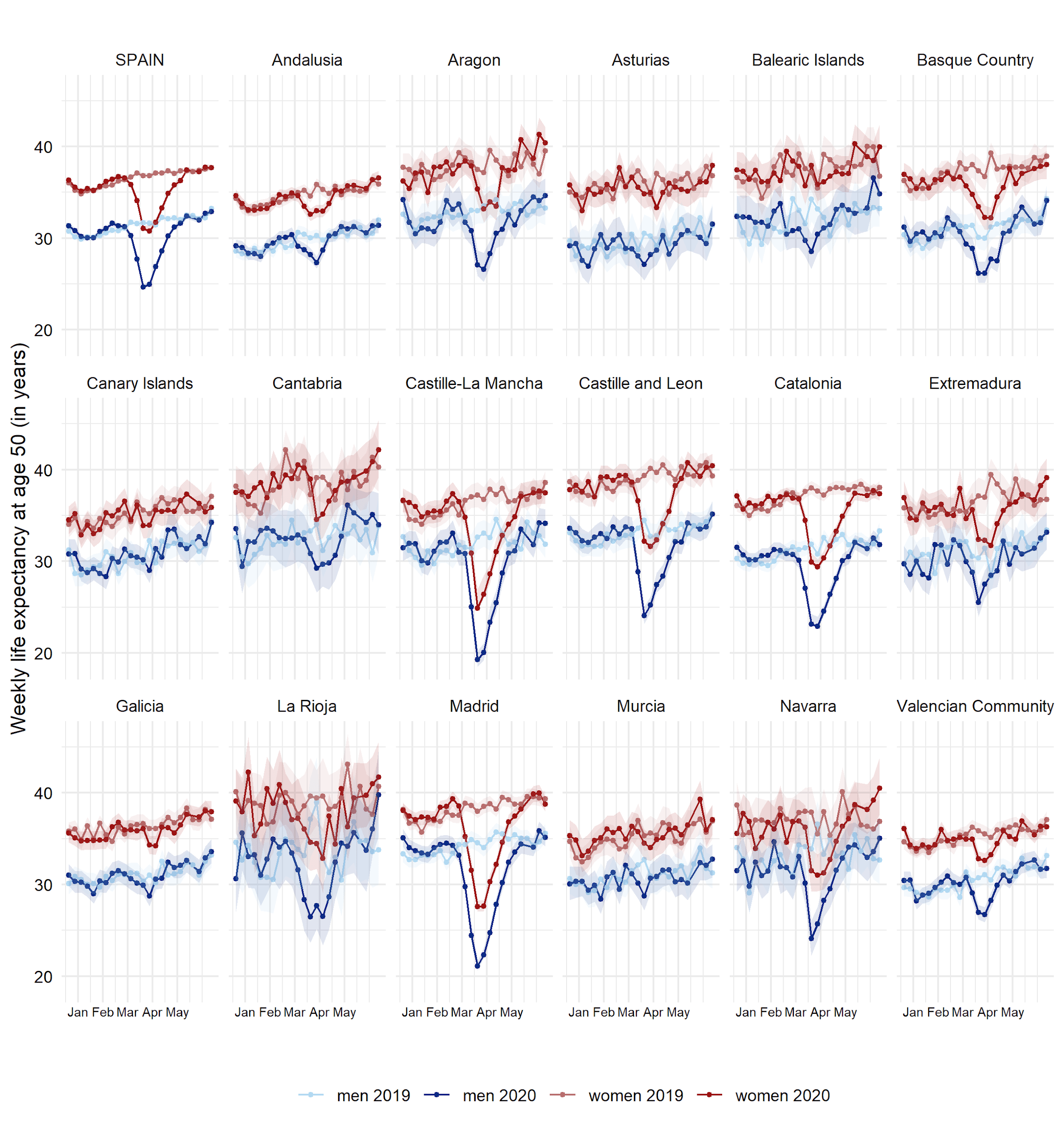


**Appendix II: Spanish MoMo coverage and implications for this study**

The Spanish daily mortality data that we have used has an overall coverage of ~93% of the population. We have done several sensitivity analyses to assess the robustness of our results:

1. Comparison between MoMo coverage and estimated coverage based on data from the 1st semester of 2019 suggests that for all regions except Aragon, Cantabria, Castille and Leon, and La Rioja had a real coverage >85%. Ten out of the 17 regions had a coverage >95%. See **Figure 1** for further details.
2. Comparison of estimated annual life expectancies at birth in 2019 (using MoMo data) with those from INE 2018 suggest small differences in estimated life expectancies in 2019 compared to 2018. Regions with real coverage below 85% were the regions where the change in life expectancy is higher. This suggested potential overestimation of life expectancies in the regions with lower coverage. See **Figure 2**.
3. Combining points (1) and (2) we observe a correlation between the real coverage and the differences in life expectancy suggesting regions with low coverage to have potentially higher biases in life expectancy estimates. See **Figure 3**.
4. A sensitivity analysis correcting by undercoverage was done by multiplying death counts by (1/coverage). As this is not advised by MoMo (See here: <https://momo.isciii.es/public/momo/dashboard/momo_dashboard.html#documentacion>), we used the coverage from the 1st semester of 2019 that we estimated and showed in Appendix II Figure 1. **See Figure 4**.
5. Differences between original annual life expectancy at birth estimates and the corresponding estimates derived from the sensitivity analyses. This figure provides general insights on the extent of life expectancy overestimates. See **Figure 5**.

In conclusion, we aimed at estimating differences in life expectancies during the COVID-19 pandemic. Due to data limitations life expectancy levels may be overestimates, especially for Aragón, Cantabria, Castille and Leon, and la Rioja, and should be interpreted carefully. However, the undercoverage of the data used is unlikely to substantially affect our main outcome, the differences between life expectancies.

**Figure 1**. MoMo coverage in semester I 2019 compared with data from “Movimento Natural de la Población” (INE) by region and sex and reported MoMo coverage by region.


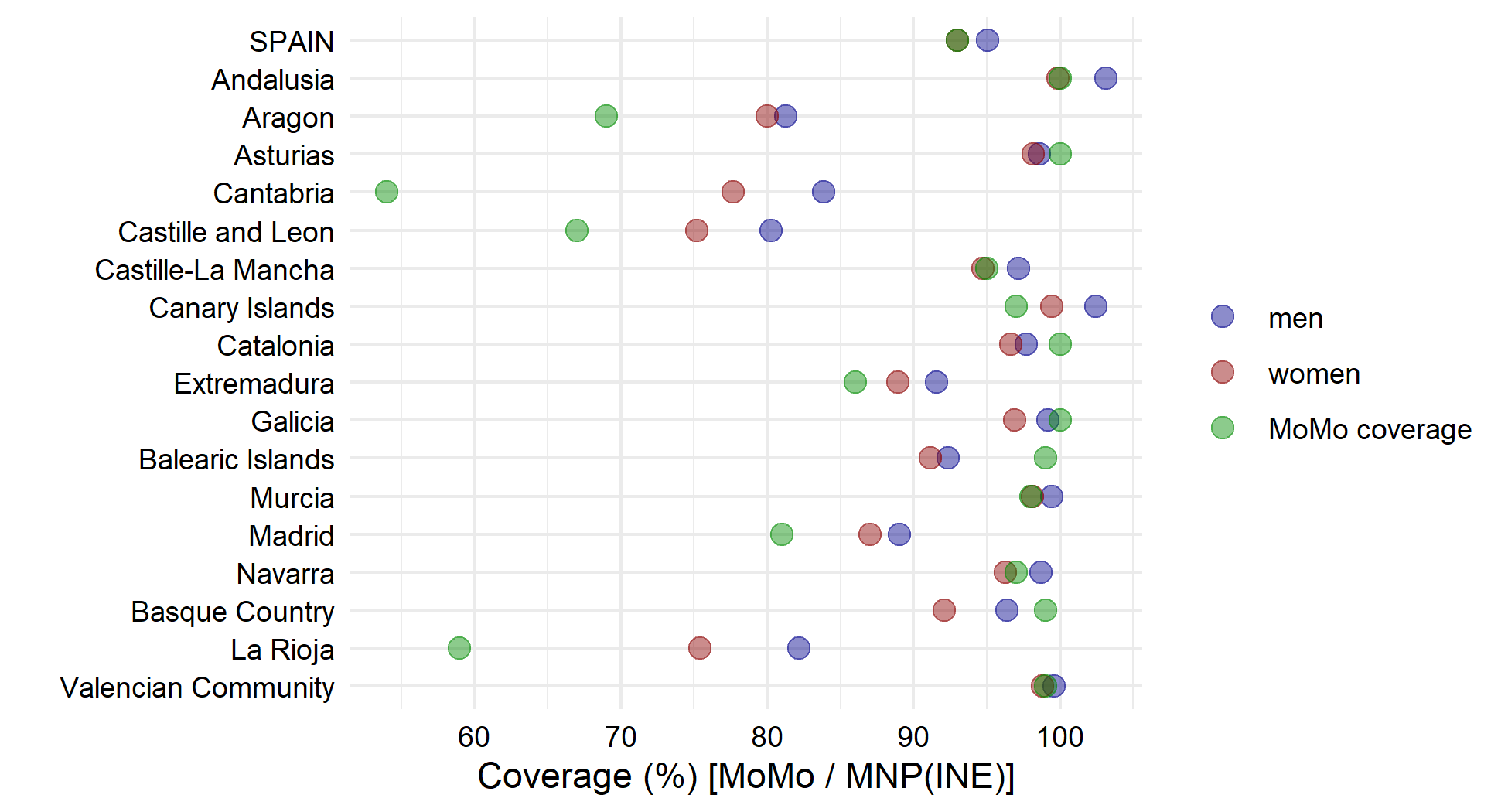


**Figure 2**. Annual life expectancy at birth in 2018 (INE) and in 2019 based on our estimates (MoMo data)


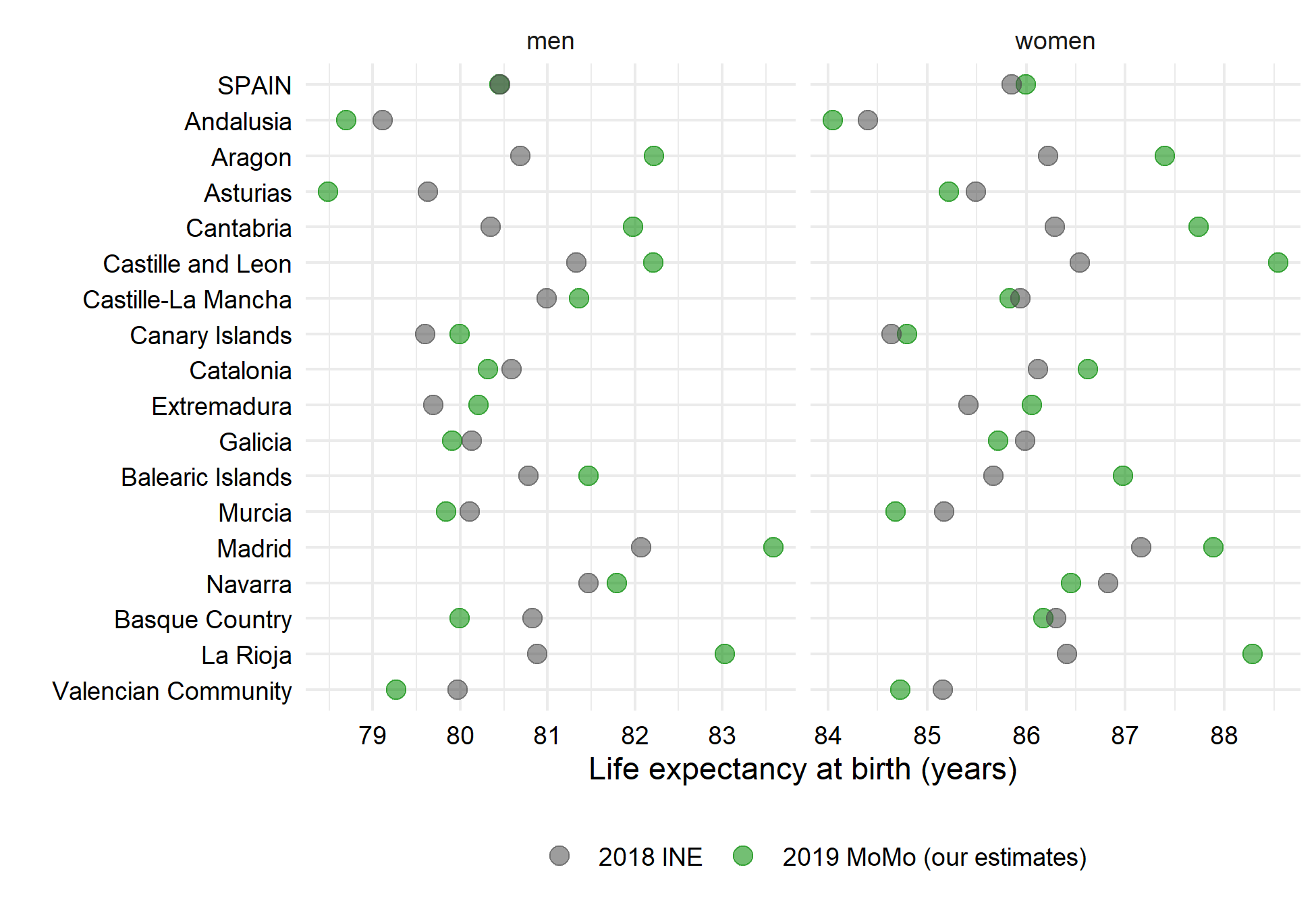


**Figure 3**. Associations between annual life expectancy at birth decline between 2018 (INE) and 2019 (own estimates, MoMo data) and coverage in MoMo


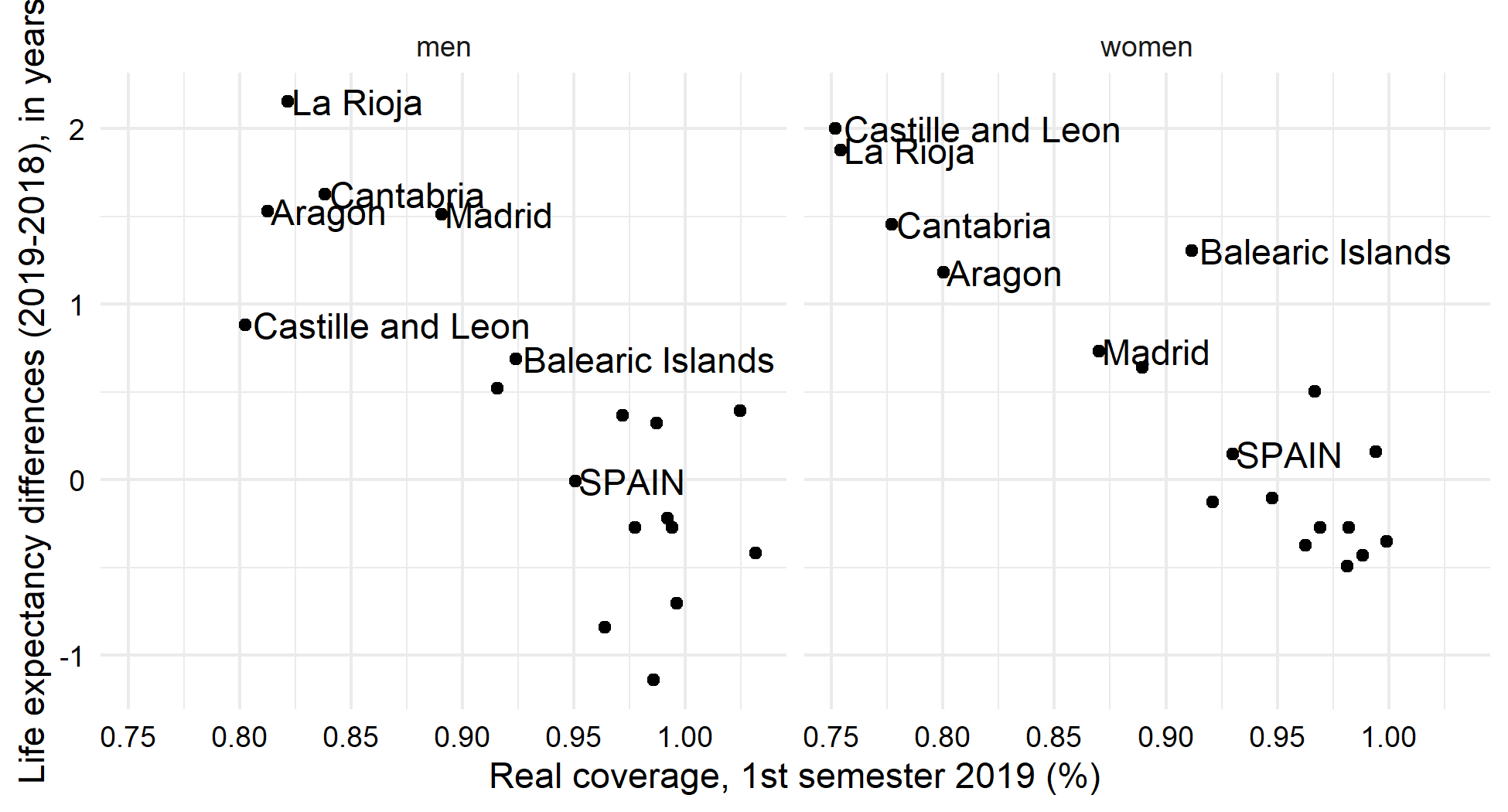


**Figure 4**. Annual life expectancy at birth in 2019, 2020* and differences between periods for Spain and its 17 regions by sex. Death counts corrected for the real coverage of MoMo*


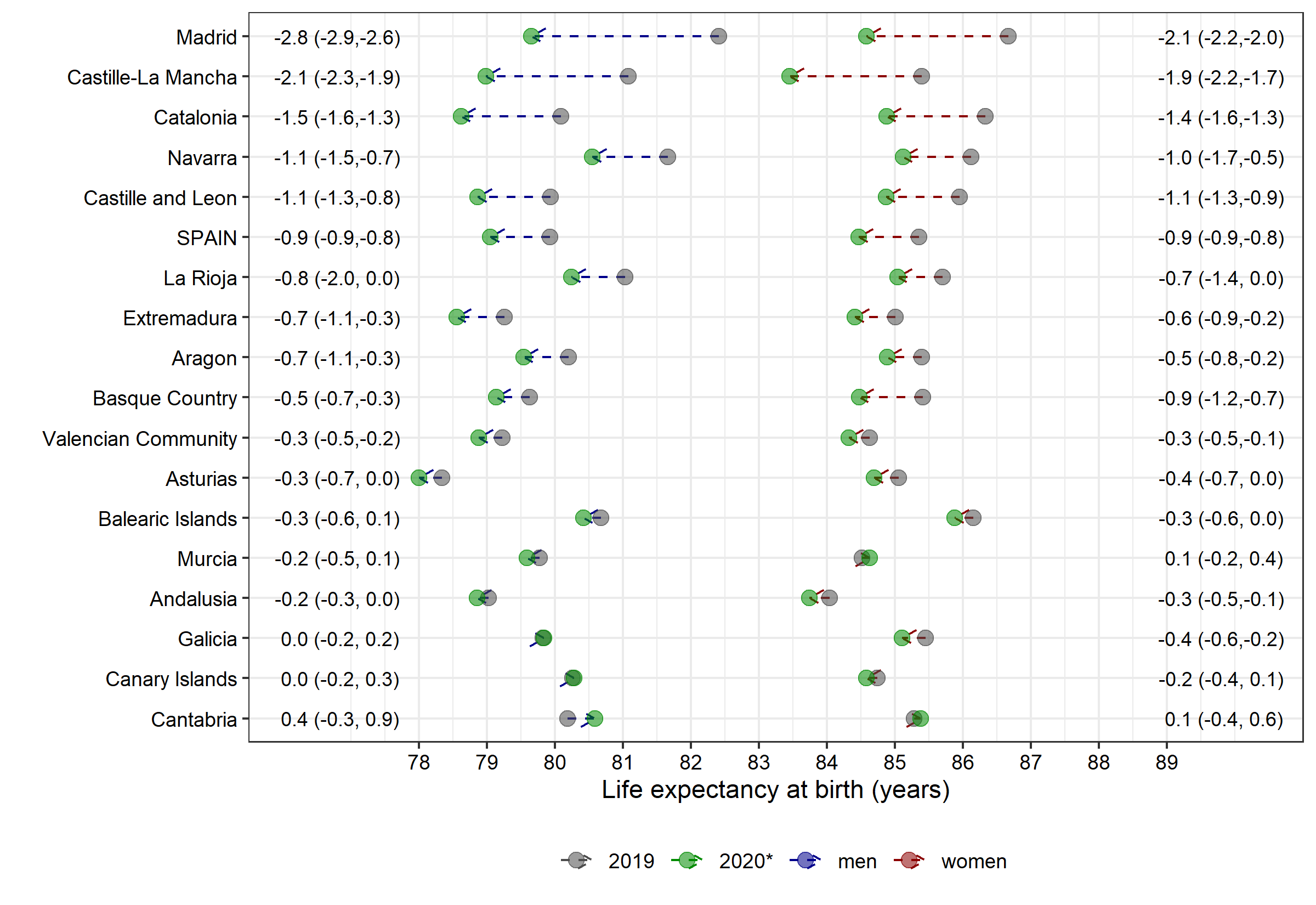


* 1st semester of 2019. Correction made using provisional death counts from Movimiento Natural de la Población (INE)

**Figure 5**. Differences in life expectancy at birth between our original estimates and the sensitivity analyses where death counts are corrected for the real coverage of MoMo


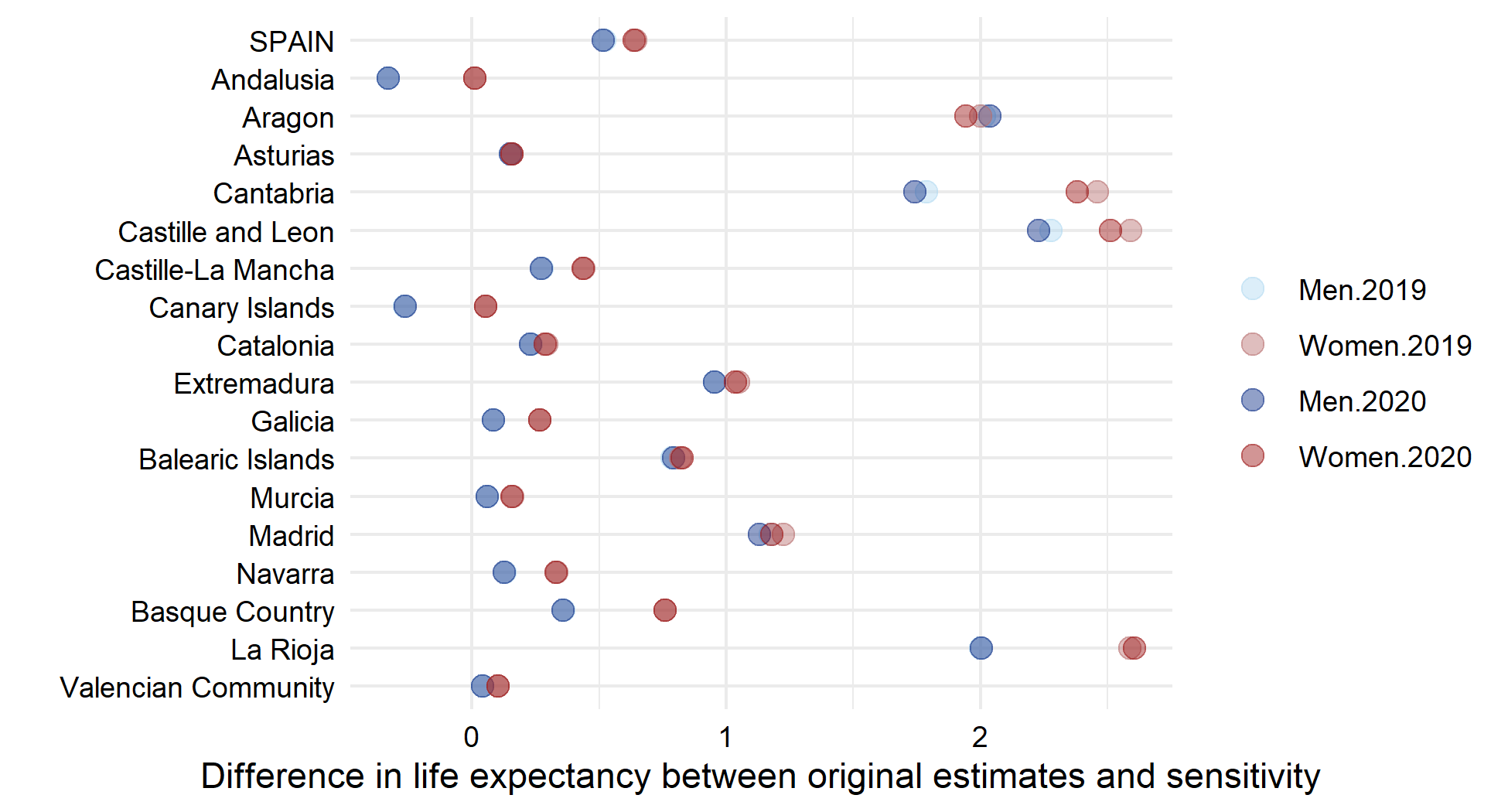

Supplement: S1 Appendix — (DOCX) [file pone.0241952.s001.docx]
