# Supplementary material for: Monitoring life expectancy levels during the COVID-19 pandemic: Example of the unequal impact of the first wave on Spanish regions
Source: PLoS One. 2020 Nov 5;15(11):e0241952. doi: 10.1371/journal.pone.0241952 (PMC7643983; doi:10.1371/journal.pone.0241952)
Supplement: Attachment — Submitted filename: Comments_PLOS.pdf [file pone.0241952.s002.pdf]

Review of the manuscript draft

"Monitoring life expectancy levels during the COVID-19 pandemic: Example of the unequal impact in Spanish regions"

by Sergi Trias-Llimós, Tim Riffe and Usama Billal

Overview

- Objectives. The authors evaluate the provisional human cost of the Covid-19 pandemic in Spanish regions by estimating and comparing weekly and annual life expectancies in 2020 vs 2019. Life expectancy allows them to provide a concise and immediate measure of the provisional death toll of the pandemic in Spain. By conducting the analysis at the sub-national level, they are able to investigate the spatially heterogeneous impact of Covid-19 across the national territory.

- Data and Methods. The authors use daily sex-specific death counts data, aggregated over broad age groups (<65, 65-75, 75+), provided by the Spanish MoMo, relative to years 2019 and 2020. They redistribute such deaths counts over 5-year age groups, applying proportions based on regional death counts relative to year 2018 provided by INE. They estimate region-, sex-, and age-specific mortality rates using the 2019 mid-year population as denominator for annual estimates, and the same population divided by 365/7 for weekly estimates. They use such rates to derive life expectancy estimates through conventional life table methods and compute the differences between 2020 and 2019 estimates.

- Results. The authors document substantial heterogeneity in the death toll of Covid-19 across Spanish regions. In Madrid, the most affected region, the sharpest drop in weekly life expectancy (11.2 to 14.8 years) is recorded in weeks 13-14, while the estimated drop in annual life expectancy is 2.8 years in the case of men and 2.1 years in the case of women. In contrast, least affected regions display no major disruption in weekly and annual life expectancy.

- Main contribution. The authors provide previously unavailable evidence on the provisional human cost of the Covid-19 pandemic in Spain, one of the most affected countries in Europe so far, and on its heterogeneity over the national territory.

- Main strengths

1. Use of overall death counts from all causes allows to capture the full impact of the Covid-19 pandemic, without suffering from issues associated to official Covid-19 deaths statistics (e.g. underreporting, exclusion of indirect mortality from other pathologies due to treatment delay, etc.).
2. Focus on regions allows to better uncover the mortality consequences of Covid-19. Indeed, given the spatial clustering of the pandemic, national figures tend to underestimate the impact on public health.
3. Likewise, use of daily death counts allow to derive weekly life expectancies which provide a timely measure of Covid-19 death toll.

- Main weaknesses
  1. Use of daily death counts originally aggregated over broad age groups (<65, 65-75, 75+) prevents a finer analysis of contribution of various age-groups to overall mortality. In fact, the disaggregation into finer ager groups as described by the authors is somewhat problematic (see below).
  2. Little discussion about the impact on men vs women and across age groups.
  3. Some methodological aspects about computation of annual mortality rates are not clear, i.e. the reference period and the population used as denominator (see below).

Major comments

1. Disaggregation of daily death counts from broad age groups (<65, 65-75, 75+) into 5-year age groups. By applying standard proportions derived from the 2018 regional death counts, the authors may introduce a bias in the derivation of age-specific mortality rates for 2020. Indeed, mortality due to Covid-19 has largely concentrated among people aged 50+ (at least so far). If most of the increase in 2020 deaths comes from middle-aged/elderly people, applying proportions based on 2018 death counts may result into an over-assignment of deaths to younger age groups, and consequently an over-estimation of the drop in life expectancy. The authors should mention and discuss this issue.

2. There is little discussion on the difference in mortality between men and women. Results from other countries suggest that mortality among men is disproportionately higher than among women, at all ages (see, for instance, Modig and Ebeling, 2020; Ghislandi et al., 2020). The authors do not discuss this point in the manuscript. In fact, they find that the estimated drop in (annual) life expectancy is remarkably similar for men and women across most Spanish regions (Figure 1). This point deserves further attention. In particular, the authors should discuss why the mortality pattern of Covid-19 in Spain is not as gendered as in other European countries.

3. It is not entirely clear which is the period over which the shifted life expectancy (2020*) is computed. In line 86, they write *"[…] up to 5 July, 2020".* In line 216, they refer to the period "*[…] May 2019 to May 2020 […]*". This point should be clarified, possibly in the Methods section.

4. It is not entirely clear which population measure is used as denominator in the computation of age-specific mortality rates for shifted annual estimates. The authors state that they use 2019 mid-year population for annual estimates. Do they use it also to compute annual life expectancy between May 2019 and May 2020, mentioned in line 216? If yes, does this potentially affect their estimates in a substantial way?

Minor comments

1. There is little discussion on the mortality impact of Covid-19 over different age groups. Since life expectancy is a summary of age-specific mortality rates, which weighs young-age deaths more than old-age deaths, the authors may

stress that the estimated drops in life expectancy are particularly remarkable if one considers that deaths have (probably) occurred mostly at older ages.

2. Page 3, lines 43-44: a reference period should be added to the statement "*Spain has been one of the most affected countries with more than 28,000 deaths with laboratory confirmation of COVID-19*" (28,000 deaths to which date?).

3. Page 5, line 85: should be clarified that most available weekly data refer to current year (i.e. week 27 of 2020).

4. Page 9, lines 148-150: the meaning of the sentence "*The little available evidence […], nearly 2 years"* is not clear. Do the authors mean that the impact of Covid on LE in Spain is larger than in England & Wales, or the contrary?

5. Page 12, line 216: the 2.8 year drop is for men? It should be specified.

6. Page 18, line 336: there is probably a typo in the sentence *"that up to…"*.

7. Lines 151 and 170: the authors employ the adjective *"elevated"*. I am not sure this adjective is used in English. Why not increased/high/higher?

8. Figure 1: why does the confidence bands for weekly LE in 2020 get much stricter (seem to disappear) for Catalonia and Madrid between March and April? This comment applies for the same regions also to Figure S4

9. Figure 2 and all Figures analogous to Figure 2: the legend is not straightforward. Maybe, the authors can use different symbols for changes in life expectancy (arrows) and point estimates (circles).

10. Figure 2: for the sake of clarity, the authors may specify which gender the columns with changes in LE and CIs refer to.

11. Figures S1: why there are not confidence bands?

12. Figure 2 in Appendix II: it could be useful to add confidence intervals to point estimates to show in which cases the two estimates are different in a statistically significant way.
