# Supplementary material for: Monitoring life expectancy levels during the COVID-19 pandemic: Example of the unequal impact of the first wave on Spanish regions
Source: PLoS One. 2020 Nov 5;15(11):e0241952. doi: 10.1371/journal.pone.0241952 (PMC7643983; doi:10.1371/journal.pone.0241952)
Supplement: Attachment — Submitted filename: Second_review_PLOS.pdf [file pone.0241952.s004.pdf]

Second review of the manuscript draft

"Monitoring life expectancy levels during the COVID-19 pandemic: Example of the unequal impact in Spanish regions"

by Sergi Trias-Llimós, Tim Riffe and Usama Billal

The authors were able to effectively address the major issues pointed out in the first review. In particular:

- They were able to provide a convincing answer to the issues related to the use of age-specific mortality rates from 2018.
- They were able to provide some convincing explanations for why the mortality impact of the first wave of Covid-19 in Spain is not as gendered as in other European countries.

The authors can find comments relative to some minor issues below.

Minor comments

1. <u>Disaggregation of daily death counts from broad age groups (<65, 65-75, 75+) into 5-year age groups</u>. In the updated text, the authors *implicitly* acknowledge that using 2018 age-specific mortality rates could be problematic. For this reason, they show that there is a strong correlation (>0.9) between their estimates of LE drop and estimates of LE drop based on provisional age-specific mortality rates provided by INE. This evidence suggests that their estimates are, indeed, robust. Still, I think that for sake of research quality, the authors should *explicitly* state in the text why using age-specific mortality rates from 2018 could be problematic (i.e. that it may lead to over-estimation of LE drop).

2. Figure 2 and all Figures analogous to Figure 2: the legend is still not straightforward. In their reply to the reviewer, the authors say that they have removed the arrows, but in the updated version of the paper, there are still arrows in Figure 2, which seems identical to the one attached to the first version of the paper.

3. Figure 2: reiterating the comment from the first review round, the authors should specify which gender the columns with changes in LE and CIs refer to. In their reply to the reviewer, the authors say that they have labelled the columns, but in Figure 2 of the updated paper, columsn are not labelled.
