# Supplementary material for: Monitoring life expectancy levels during the COVID-19 pandemic: Example of the unequal impact of the first wave on Spanish regions
Source: PLoS One. 2020 Nov 5;15(11):e0241952. doi: 10.1371/journal.pone.0241952 (PMC7643983; doi:10.1371/journal.pone.0241952)
Supplement: Attachment — Submitted filename: Reply to the reviewers.docx [file pone.0241952.s003.docx]

Reviewer #1: Review of the manuscript draft
“Monitoring life expectancy levels during the COVID-19 pandemic: Example of the unequal impact in Spanish regions”
by Sergi Trias-Llimós, Tim Riffe and Usama Billal

Overview

- Objectives. The authors evaluate the provisional human cost of the Covid-19 pandemic in Spanish regions by estimating and comparing weekly and annual life expectancies in 2020 vs 2019. Life expectancy allows them to provide a concise and immediate measure of the provisional death toll of the pandemic in Spain. By conducting the analysis at the sub-national level, they are able to investigate the spatially heterogeneous impact of Covid-19 across the national territory.

- Data and Methods. The authors use daily sex-specific death counts data, aggregated over broad age groups (<65, 65-75, 75+), provided by the Spanish MoMo, relative to years 2019 and 2020. They redistribute such deaths counts over 5-year age groups, applying proportions based on regional death counts relative to year 2018 provided by INE. They estimate region-, sex-, and age-specific mortality rates using the 2019 mid-year population as denominator for annual estimates, and the same population divided by 365/7 for weekly estimates. They use such rates to derive life expectancy estimates through conventional life table methods and compute the differences between 2020 and 2019 estimates.

- Results. The authors document substantial heterogeneity in the death toll of Covid-19 across Spanish regions. In Madrid, the most affected region, the sharpest drop in weekly life expectancy (11.2 to 14.8 years) is recorded in weeks 13-14, while the estimated drop in annual life expectancy is 2.8 years in the case of men and 2.1 years in the case of women. In contrast, least affected regions display no major disruption in weekly and annual life expectancy.

- Main contribution. The authors provide previously unavailable evidence on the provisional human cost of the Covid-19 pandemic in Spain, one of the most affected countries in Europe so far, and on its heterogeneity over the national territory.

- Main strengths
1. Use of overall death counts from all causes allows to capture the full impact of the Covid-19 pandemic, without suffering from issues associated to official Covid-19 deaths statistics (e.g. underreporting, exclusion of indirect mortality from other pathologies due to treatment delay, etc.).
2. Focus on regions allows to better uncover the mortality consequences of Covid-19. Indeed, given the spatial clustering of the pandemic, national figures tend to underestimate the impact on public health.
3. Likewise, use of daily death counts allow to derive weekly life expectancies which provide a timely measure of Covid-19 death toll.


- Main weaknesses
1. Use of daily death counts originally aggregated over broad age groups (<65, 65-75, 75+) prevents a finer analysis of contribution of various age-groups to overall mortality. In fact, the disaggregation into finer ager groups as described by the authors is somewhat problematic (see below).
2. Little discussion about the impact on men vs women and across age groups.
3. Some methodological aspects about computation of annual mortality rates are not clear, i.e. the reference period and the population used as denominator (see below).


Major comments

1. The disaggregation of daily death counts from broad age groups (<65, 65-75, 75+) into 5-year age groups presents some issues. By applying standard proportions derived from the 2018 regional death counts, the authors may introduce a bias in the derivation of age-specific mortality rates for 2020. Indeed, mortality due to Covid-19 is largely concentrated among people aged 50+ (at least so far). If most of the increase in 2020 deaths comes from middle-aged/elderly people, applying proportions based on 2018 death counts may result into an over-assignment of deaths to younger age groups, and consequently an over-estimation of the drop in life expectancy. The authors should mention and discuss this issue.

We thank the reviewer for this important comment. This is an important issue as the age-pattern of deaths may differ between 2018 and first half of 2020.

Recent age-specific mortality estimates have been released by INE (provisional estimates). These estimates rely on the same source of the MoMo data and are corrected by undercoverage (in a similar way to ours in Appendix II), but its correction is not age-specific. Even in that case, we believed that it would be ideal to compare the main estimates derived from this source, which confirms the magnitude of the life expectancy loss between 2019 and 2020* (see Figure 4). Following the reviewer advice, we have added this figure in the main paper and discuss this issue in the discussion:

“In our analyses we have assumed the age pattern of deaths in 2018 applies to the first half of 2020 (2020*). In a sensitivity analysis we used age-specific mortality estimates recently released by the INE, corrected by the undercoverage of electronically reported mortality data (~7%) [30], and we obtained similar estimates on the life expectancy gaps between 2019 and 2020* (Figure 4). Thus, this confirms that our results are robust irrespective of the data source and underlying assumptions”.

2. There is little discussion on the difference in mortality between men and women. Results from other countries suggest that mortality among men is disproportionately higher than among women, at all ages (see, for instance, Modig and Ebeling, 2020; Ghislandi et al., 2020). The authors do not discuss this point in the manuscript. In fact, they find that the estimated drop in (annual) life expectancy is remarkably similar for men and women across most Spanish regions (Figure 1). This point deserves further attention. In particular, the authors should discuss why the mortality pattern of Covid-19 in Spain is not as gendered as in other European countries.

In some of the most affected regions this sex-gap is observed. However, this is not the case in other regions, nor in the whole country. We have now included a paragraph dealing with this issues and hypothesising potential explanations for the lack of sex-differences that has been seen in other populations.

“Interestingly, our results suggest a similar mortality drop for men and women (0.9 years, in annual life expectancy). This finding contrasts with estimates from Italy, Sweden, and England and Wales, where the largest life expectancy drops –although of different magnitude- were observed for men compared to women (7–9). Whereas a typical sex-gap was observed in some of the most affected regions (e.g. Madrid or Castille-La Mancha), it was not observed in some of the least affected regions (in Figure 2: Cantabria 0.4 years for men and 0.0 for women; Galicia 0.0 years for men and -0.3 years for women; or Andalusia -0.2 years for men and -0.3 years for women). Although this may be counterbalancing the national results, it is unlikely to entirely explain the observed similar life expectancy drop between men and women. Looking for explanations of this rather comparatively high mortality among women different factors may be at play. First, Spanish women have the highest life expectancy in Europe (26), but rank 9^th^ in healthy life expectancy at age 65, indicating a higher rate of comorbidities and potentially a higher vulnerability to COVID-19 (27). In fact, while life expectancy at birth in 2018 was 5.6 years higher in Spanish women as compared to men, healthy life expectancy at age 65 was 0.2 years lower in women compared to men. Second, nursing homes, mostly populated by women (28), were particularly affected by COVID-19 in Spain (19,26). Further potential explanations for this gap would require analysing cause-of-death and place-of-death mortality data, which is unfortunately not available for Spain at the time of writing”.

3. It is not entirely clear which is the period over which the shifted life expectancy (2020*) is computed. In line 86, they write “[…] up to 5 July, 2020”. In line 216, they refer to the period “[…] May 2019 to May 2020 […]”. This point should be clarified, possibly in the Methods section.

We appreciate the reviewer comment. The text referring to “May 2019 to May 2020” was incorrect. We have now corrected it as follows:

“… while the annual life expectancy for the one year window that closes out on 5 July 2020 (week 27)…”.

4. It is not entirely clear which population measure is used as denominator in the computation of age-specific mortality rates for shifted annual estimates. The authors state that they use 2019 mid-year population for annual estimates. Do they use it also to compute annual life expectancy between May 2019 and May 2020, mentioned in line 216? If yes, does this potentially affect their estimates in a substantial way?

We have corrected the text in (previous) line 216 as we refer to the one year window that closes out on 5 July 2020. We have used 2019 mid-year population as this was the latest available estimates. Ideally, we should have used data on 1 January 2020. We do not believe that using population estimates of have a year older would affect our final estimates. As we analysed data in 5-year age groups, most of the mid-2019 population estimates (~90%) are well allocated within each age group. Therefore, we do not believe this could have an appreciable impact on our results.

Minor comments

1. There is little discussion on the mortality impact of Covid-19 over different age groups. Since life expectancy is a summary of age-specific mortality rates, which weighs young-age deaths more than old-age deaths, the authors may stress that the estimated drops in life expectancy are particularly remarkable if one considers that deaths have (probably) occurred mostly at older ages.

We have now acknowledged that most of the deaths occurred at adult and older ages, which have a lower impact on life expectancy. Given that, the observed drops are remarkable.

2. Page 3, lines 43-44: a reference period should be added to the statement “Spain has been one of the most affected countries with more than 28,000 deaths with laboratory confirmation of COVID-19” (28,000 deaths to which date?).

We have now rewritten the text to make it clear we refer to July 19^th^ 2020:

“According to WHO, 2020 nearly 600,000 confirmed COVID-19 deaths occurred worldwide by July 19th 2020 (1). Spain was one of the most affected countries with more than 28,000 deaths with laboratory confirmation of COVID-19 by that date (1)”.

3. Page 5, line 85: should be clarified that most available weekly data refer to current year (i.e. week 27 of 2020).

4. Page 9, lines 148-150: the meaning of the sentence “The little available evidence […], nearly 2 years” is not clear. Do the authors mean that the impact of Covid-19 on LE in Spain is larger than in England & Wales, or the contrary?

We have now amended this sentence accordingly:

“The little available evidence on similar studies for other populations suggests that, at the country level, this impact may be larger in England & Wales, nearly 2 years (8)”.

5. Page 12, line 216: the 2.8 year drop is for men? It should be specified.

We have now explicitly add in the text that we refer to men.

6. Page 18, line 336: there is probably a typo in the sentence “that up to…”.

We have removed “that” to correct the typo: “…from the one year window up to 5 July 2020”.

7. Lines 151 and 170: the authors employ the adjective “elevated”. I am not sure this adjective is used in English. Why not increased/high/higher?

We have now used the adjective “increased”.

8. Figure 1: why does the confidence bands for weekly LE in 2020 get much stricter (seem to disappear) for Catalonia and Madrid between March and April? This comment applies for the same regions also to Figure S4

The confidence intervals remain relatively stable along the weeks (Men, around 1.7-1.9 in Catalonia and 1.9-2.2 in Madrid). The visual impression of narrower CI in march and April is seen due to the drop in life expectancy.

9. Figure 2 and all Figures analogous to Figure 2: the legend is not straightforward. Maybe, the authors can use different symbols for changes in life expectancy (arrows) and point estimates (circles).

We have removed the arrows as they were confusing, especially for those regions in which life expectancy differences are lower.

10. Figure 2: for the sake of clarity, the authors may specify which gender the columns reporting changes in LE and CIs refer to.

We have now labelled the columns to improve the clarity of the plot.

11. Figure S1: why there are not confidence bands?

Confidence intervals were now added to this figure.

12. Figure 2 in Appendix II: it could be useful to add confidence intervals to point estimates to show in which cases the two estimates are different in a statistically significant way.

Appendix II aimed to provide different sort of estimates to validate our main results as the coverage of the data we have used was not 100%, but 93%. In Figure 2 we used life expectancy estimates from INE 2018, where confidence intervals were not provided. To homogenize the figure, we did not show the confidence intervals for our estimates. Furthermore, in the plot estimates from 2019 are compared with the 2018 data, which add another layer of complexity when interpreting the differences. The main objective of the figure was to show that life expectancy estimates are quite close to each other for the regions with coverage <95%, but tend to differ more in the regions with low coverage. We believe that Figure 2 in Appendix II suffices for this.

Reviewer #2: The paper provides a very straightforward and relevant assessment on the impact of COVID-19 on life expectancy in Spain and its regions based on daily count of deaths. Overall, I have very positive feedback and would recommend publication after fixing some small issues.

1-In the calculation of the yearly life expectancy, what is assumed for the rest of the year (past week 20)? First, I thought you assume that the mortality is back to normal from week 20 until the end of the year, as you generally wrote “life expectancy in 2020”. When I read the last 2 paragraphs, I understood that the yearly life expectancy is indeed from May 2019 to May 2020 (thus no forecast) and not an estimate for 2020. On line 329, you then write that the period is until July 2020 (which would imply a small forecast). This is a bit confusing.

We refer to life expectancy in 2020* as the life expectancy in the annual period that closes on 1 July 2020. Therefore, no assumptions for the future were applied. We have now made sure that this was more clearly stated in the text, and we have corrected a wrong piece of text (from an earlier version) stating that it was from May 2019 to May 2020.

I would specify in the objectives that you assess the impact of the first wave of COVID-19 (from week 11 to 20) on life expectancy (otherwise, with the second wave that is rising, the paper might be quickly outdated). I also suggest being clearer in the method about the period used as reference in the yearly life expectancy. If you did some forecasts for the rest of the year, specify it more clearly.

We thank the reviewer for this important insight. Indeed, we have now referred to the first wave of the COVID-19 both in the objective and in the title, so the paper can clearly by understood in the coming months and years.

As mentioned above, we have now made sure that the definition of “2020*” (annual period that closes up in 5 July 2020).

2-Because things are evolving very quickly, I would be careful with statements such as “one of the most severely affected countries in the world”(142, 182 and many other places). In February, the most severely affected country was China. 5 months later, China was among the least affected countries per inhabitant. The situation can thus be radically different just a few weeks after the paper is published.

We have now explicitly mentioned in these sentences refer to the situation in the first half of 2020. Thus, we ensure that the text is precisely read and understood in the coming weeks and years.

Similarly, authors should revise statements that are already outdated such as (196-198) “Other populations, for example Brazil, Ecuador or Chile are somewhat behind European countries in the pandemic at the time of this writing, but rapidly experiencing a dramatic death toll increase (24).”

We have now rephrased this sentence to make it clearer over time:

“At the time of writing, other populations, for example Brazil, Ecuador or Chile have also been substantially affected by COVID-19 (25). If the age distribution of deaths in other populations skews younger, this may lead to stronger impacts on life expectancy in these populations”.

Minor.
Lines 126, 131 and some other places: there are *, but it’s not clear what they stand for. I see on lines 324, 329 and 335 different meanings.

We have now clarified in the figures title and in figures notes what the * meant. To avoid confusion between 2020* (annual period up to week 27 2020), we have now referred to figures notes with superscripts instead of with “*”.
